# Retinal thickness as potential biomarker in posterior cortical atrophy and typical Alzheimer’s disease

**DOI:** 10.1186/s13195-019-0516-x

**Published:** 2019-07-18

**Authors:** Jurre den Haan, Lajos Csinscik, Tom Parker, Ross W. Paterson, Catherine F. Slattery, Alexander Foulkes, Femke H. Bouwman, Frank D. Verbraak, Philip Scheltens, Tunde Peto, Imre Lengyel, Jonathan M. Schott, Sebastian J. Crutch, Timothy J. Shakespeare, Keir X. X. Yong

**Affiliations:** 1 Department of Neurology, Amsterdam Neuroscience, Alzheimer Center Amsterdam, Amsterdam UMC, Vrije Universiteit Amsterdam, Mailbox 7057, 1007 MB Amsterdam, The Netherlands; 2 Centre for Experimental Medicine, Queen’s University, Belfast, UK; 3 Institute of Ophthalmology UCL, London, UK; 4 Dementia Research Centre, UCL Queen Square Institute of Neurology, London, UK; 5 NIHR Biomedical Research Centre, Moorfields Eye Hospital NHS Foundation Trust and UCL, London, UK; 6 Department of Ophthalmology, Amsterdam UMC, Vrije Universiteit, Amsterdam, The Netherlands

**Keywords:** Alzheimer’s disease, Posterior cortical atrophy, Optical coherence tomography, Retinal thickness, Biomarker, MRI

## Abstract

**Background:**

Retinal thickness can be measured non-invasively with optical coherence tomography (OCT) and may offer compelling potential as a biomarker for Alzheimer’s disease (AD). Retinal thinning is hypothesized to be a result of retrograde atrophy and/or parallel neurodegenerative processes. Changes in the visual pathway are of particular interest in posterior cortical atrophy (PCA), the most common atypical AD phenotype predominantly affecting the parietal-occipital cortices. We therefore evaluated retinal thickness as non-invasive biomarker of neurodegeneration in well-characterized participants with posterior cortical atrophy (PCA) and typical Alzheimer’s disease (tAD).

**Methods:**

Retinal thickness measures were acquired from 48 patient participants (*N* = 25 PCA; *N* = 23 tAD) fulfilling consensus diagnostic criteria and 70 age-matched controls. Participants were recruited between 2014 and 2016. All participants underwent optical coherence tomography (OCT) imaging, including measurement of peripapillary retinal nerve fiber layer (pRNFL) thickness and total macular thickness (mRT). Participants did not show evidence of any significant ophthalmological conditions. Subgroup analyses were performed in participants with available MRI and CSF measures, providing evidence of neurodegeneration and underlying AD pathology respectively.

**Results:**

There was no evidence of overall between-group differences in pRNFL thickness (mean PCA 98.7 ± 12.2; tAD 99.9 ± 8.7; controls 99.6 ± 10.0 μm, one-way analysis of variance (ANOVA) *p* = 0.92) or total mRT (mean PCA 266.9 ± 16.3; tAD 267.8 ± 13.6; controls 269.3 ± 13.6 μm, one-way ANOVA *p* = 0.75). Similarly, subgroup analysis with MRI biomarkers (PCA = 18, tAD = 17, controls = 31) showing neurodegeneration, and CSF biomarkers (PCA = 18, tAD = 14, controls = 13) supporting underlying AD pathology did not provide evidence of overall between-group differences in pRNFL or mRT measures (all *p* > 0.3).

**Conclusions:**

Retinal thickness did not discriminate tAD and PCA from controls or from one another despite unequivocal differences on standard clinical, neuro-imaging and CSF measures. Findings from this well-characterized sample, including cases with PCA, do not support the hypothesis that retinal neurodegeneration, measured using conventional OCT, is a useful biomarker for AD or PCA.

## Introduction

There is an urgent need for non-invasive Alzheimer’s disease (AD) biomarkers. The retina, sharing its embryological origin with the brain, may reflect AD hallmark pathology [1, 2]. Ocular manifestations of AD may include retinal thinning [3, 4], vascular changes [5] and amyloid-beta [6] and tau [7] retinal deposition. Retinal thinning has been proposed as a promising non-invasive imaging biomarker, purportedly mirroring cortical atrophy owing to trans-synaptic retrograde neurodegeneration, and/or reflecting parallel processes in both retinal and cortical neurons [8]. However, conflicting study findings of retinal thinning in AD [3] have prompted recommendations for investigations comprising well-characterized participants, controlling for confounding factors and comparing purported ocular biomarkers with established AD biomarkers [9].

Retinal thinning is of particular interest in posterior cortical atrophy (PCA), the canonical ‘visual dementia’, and the most common atypical presentation of AD [10]. PCA is a neurodegenerative syndrome presenting with progressive cortico-visual problems in contrast to relatively well-preserved memory, language and insight [11]. PCA preferentially affects parietal-occipital and occipito-temporal lobes, key areas for visual processing that receive input from retino-cortical projections mainly through the lateral geniculate nucleus and optical radiations [12, 13]. Consequently, PCA represents a patient group uniquely positioned to evaluate the hypothesis of retrograde atrophy from cortical visual areas following neurodegeneration.

We assessed retinal thickness measured with optical coherence tomography in PCA, typical AD (tAD) and control participants. Consistent with the hypothesis of trans-synaptic retrograde neurodegeneration, we hypothesized that particular reductions in retinal thickness would be observed in PCA relative to both tAD and control participants, and in tAD relative to control participants.

## Methods

### Participants

We enrolled 48 patients, 25 patients with posterior cortical atrophy (PCA) and 23 with typical Alzheimer’s disease (tAD), in addition to 70 cognitively healthy controls. Participants were recruited from a tertiary specialist centre, the University College London (UCL) Dementia Research Centre (DRC), between 2014 and 2016. Participant groups were well-matched for demographic characteristics, and there was no evidence of between-group differences in age or gender (Table 1). PCA and tAD patients were assessed by consultant neurologists with expertise in cognitive neurology and fulfilled consensus criteria for PCA and NIA-AA criteria for tAD respectively [11, 14]. PCA patients fulfilled Mendez et al. [15] and Tang-Wai et al. [16] proposed clinical criteria based on available information at baseline visit and expert retrospective clinical review. Controls did not show evidence of cognitive impairment as assessed by MMSE (≥ 27). PCA patients did not fulfil clinical criteria for dementia with Lewy bodies (DLB), corticobasal degeneration (CBD) or prion disease or exhibit associated clinical features (e.g. visual hallucinations, pyramidal signs, reduplicative phenomena, parkinsonism, alien limb syndrome, asymmetric dystonia and myoclonus, ataxia), and cases were therefore classified as PCA due to AD pathology. As an inclusion criterion, memory was the cognitive domain predominantly affected in tAD patients; tAD patients did not fulfil clinical criteria for logopenic variant of primary progressive aphasia [17] or frontal variant Alzheimer’s disease [18]. Exclusion criteria were a history of other neurological or major psychiatric diseases. Ethical approval was provided by the National Research Ethics Service Committee London Queen Square; all participants provided written informed consent.

**Table 1:** Demographic characteristics and proposed and established biomarker data for PCA, tAD and control groups

Demographics		Posterior cortical atrophy	Typical Alzheimer’s disease	Controls	*p* value			
Number		25	23	70				
Sex (m/f)		11/14	14/9	29/41	0.26^a^			
Age		67.0 (±7.1)	64.5 (±6.8)	66.3 (±7.7)	0.47^b^			
MMSE		22.1 (±5.4)	19.8 (±5.6)	29.5 (±0.8)	*< 0.001* ^c^			
Biomarkers		Posterior cortical atrophy	Typical Alzheimer’s disease	Controls	Linear regression models^c^			
					PCA		tAD	
					Beta	*p* value	Beta	*p* value
OCT^1^	Mean peripapillary retinal nerve fibre layer thickness (pRNFL) (μm)	98.8 (±12.2)	99.9 (±8.7)	99.6 (±10.0)	− 0.02	0.80	− 0.01	0.89
	Mean macular retinal thickness (mRT) (μm)	266.9 (±16.3)	267.8 (±13.6)	269.3 (±13.6)	− 0.07	0.50	− 0.09	0.38
MRI subset^2^	AD Signature Thickness (mm)	2.5 (±0.2)	2.5 (±0.2)	2.8 (±0.1)	− 0.73	*< 0.001*	− 0.60	*< 0.001*
	Hippocampus volume (mm^3^)	6631.4 (±713.9)	6411.6 (±1072.1)	7847.8 (±873.5)	− 0.44	*< 0.001*	−0.61	*< 0.001*
	PCA signature thickness (mm)	1.7 (±0.2)	1.9 (±0.1)	2.1 (±0.1)	− 0.78	*< 0.001*	− 0.35	*< 0.001*
	Estimated intracranial volume (*10^6^ mm^3^)	1.5 (±0.1)	1.5 (±0.2)	1.5 (±0.2)	−0.14	0.21	−0.15	0.17
CSF subset^3^	Aβ_-1-42_ (ng/L)	395.9 (±140.1)	300.1 (±133.2)	900.9 (±221.9)	− 0.70	*< 0.001*	− 0.90	*< 0.001*
	Tau_−181_ (ng/L)	573.4 (±306.4)	778.2 (±359.1)	265.1 (±119.2)	0.32	0.07	0.65	*< 0.001*
	Tau_−181_/Aβ_1–42_ ratio	1.6 (±1.0)	3.1 (±1.8)	0.3 (±0.1)	0.22	0.18	0.68	*< 0.001*

Overall cohort characteristics, and proposed OCT and established biomarkers (MRI, CSF) for posterior cortical atrophy (PCA), typical Alzheimer’s disease (tAD) and control groups including between-group comparisons. MRI and CSF data were available in subset cohorts

^1^OCT-imaging was available in 25 PCA cases, 23 tAD cases and 70 controls for pRNFL peripapillary ring scans and in 23 PCA cases, 22 tAD cases and 66 controls for macular scans. ^2^MRI was available in 18 PCA cases, 17 tAD cases and 31 controls. ^3^CSF was available in 14 PCA cases, 18 tAD cases and 12 controls

^a^Chi-square test, ^b^one-way ANOVA, ^c^Linear regression models assessing relationships between biomarkers (dependent) and diagnosis (independent) with controls as reference group, corrected for age, sex (and estimated intracranial volume (eTIV) for hippocampal volume). Reported betas are standardized betas. Significant results are indicated in italics.

### Ophthalmological assessment and OCT imaging

**Fig. 1:**
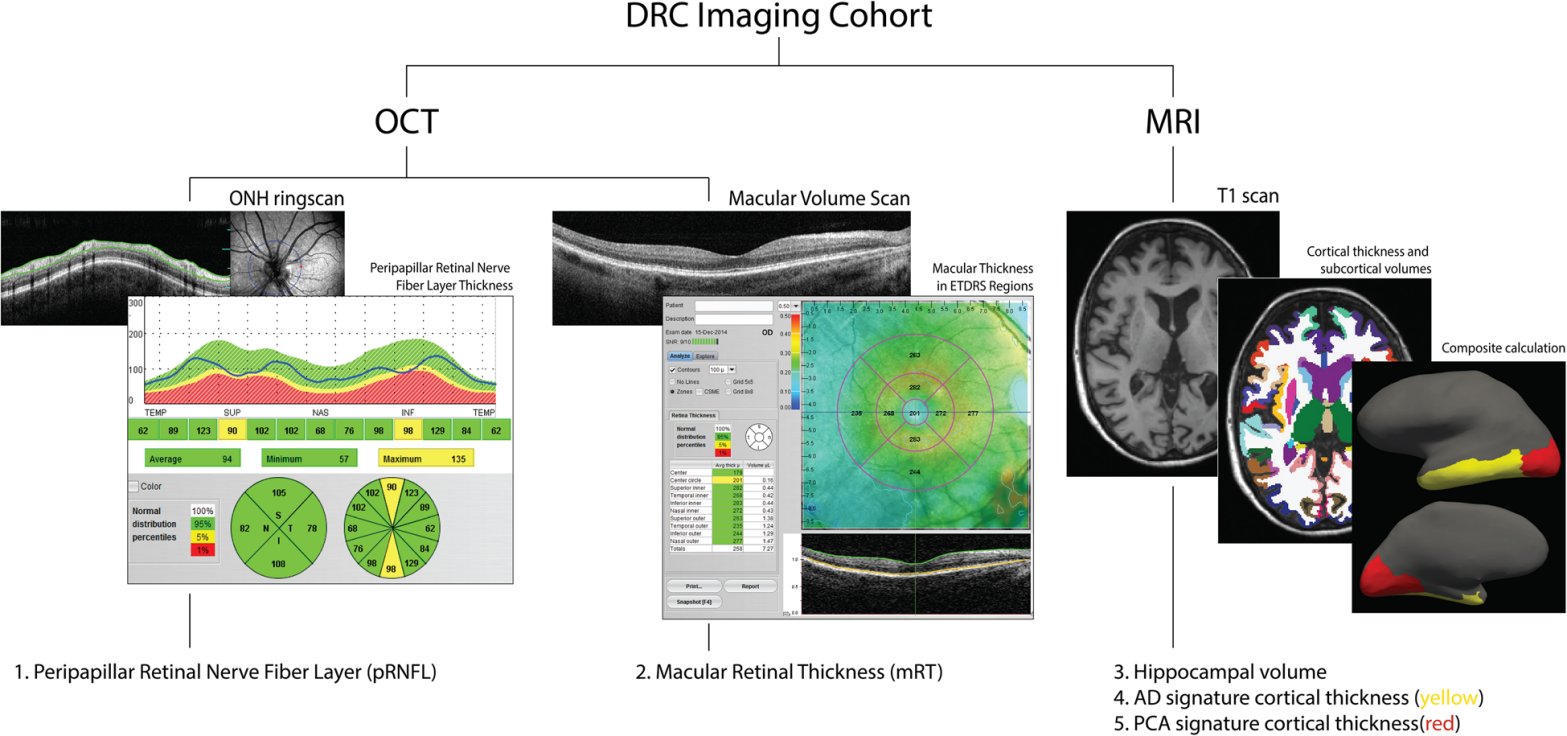
Imaging cohort. Overview of DRC imaging cohort. Abbreviations: DRC, Dementia Research Centre; OCT, optical coherence tomography; MRI, magnetic resonance imaging; ONH, optic nerve head; ETDRS, Early Treatment Diabetic Retinopathy Study

Ophthalmological history and use of medication were reviewed. Ocular exclusion criteria were a history of glaucoma or the presence of pathology that could affect retinal thickness such as glaucoma, retinoschisis, epiretinal membrane, age-related macular degeneration, hypertensive and diabetic retinopathy and retinal microcysts. All participants underwent non-midriatic optical coherence tomography with a spectral domain Optos OCT/SLO and included the following two protocols: (i) peripapillary ring scan (diameter of 12°, average of 3 b-scans, centred on the optic disk with live tracking), to measure peripapillary retinal nerve fibre layer (pRNFL) thickness (measured between vitreo-retinal interface and the outer boundary of the RNFL) and (ii) radial macular scan (diameter of 30°, 6 high-resolution b-scans centred on the fovea) to measure macular retinal thickness (mRT) (measured between vitreo-retinal interface and the mid RPE reflectance) (Fig. 1). Individual scans were quality controlled (QC) by excluding scans with a signal to noise ratio (SNR) < 6 and assessing misalignment (peripapillary ring scans) and segmentation errors. Mean pRNFL as well as in four quadrants (superior, nasal, inferior, temporal), mRT in the fovea, the inner ring [*Ø* 1–3 mm around fovea] and outer ring [*Ø* 3–6 mm around fovea] of the Early Treatment in Diabetes Retinopathy Study (ETDRS) grid were extracted. The means of both eyes (if available) were calculated for data analysis (both eyes available: peripapillary ring scan: PCA 21(84%), AD 20(87%), HC 63(90%); Macula: PCA 15(69%), AD 19(83%), HC 58(88%)). Ophthalmological assessment of OCT scans was performed by an ophthalmologist (TP), blinded to disease status. Two controls were excluded because of bilateral epiretinal membrane. For peripapillary ring scans, one tAD case failed QC. For macular scans, two PCA cases, three tAD cases and three controls failed QC.

### Magnetic resonance imaging (MRI)

A subset of participants (PCA = 18; tAD = 17; controls = 31) underwent 3-Tesla (3 T) MRI on a Siemens Magnetom Trio (Siemens, Erlangen, Germany) scanner with 32-channel phased array receiver head coil Sagittal 3D MPRAGE T1-weighted volumetric MRI sequence (TE/TI/TR = 2.9/900/2200 ms, matrix size 256 × 256 × 208, voxel size 1.1 × 1.1 × 1.1 mm). Scans were converted to Nifti format and processed using Freesurfer version 6.0.0’s cross-sectional analysis pipeline (*recon-all).* Estimates of cortical thickness for cortical regions of interest (ROIs) were extracted using the Desikan-Killiany Atlas [19], while estimates of subcortical grey matter volumes were calculated using the Fischl atlas [20] (Fig. 1). Segmented scans were visually assessed for quality control purposes following Freesurfer QC guidelines and included assessment of pial and white-matter borders and subcortical boundaries. Composite ROI’s were formed by merging anatomical labels using Freesurfer’s *mri_mergelabels* command. The following ROI’s were calculated: (1) AD signature thickness [21] (bilateral entorhinal, middle and inferior temporal and fusiform cortices), (2) bilateral hippocampal volume [22], (3) PCA signature thickness [12, 23] (bilateral lateral occipital, cuneus, pericalcarine and lingual cortices). Composite ROI’s represented areas predominantly involved in tAD (1, 2) or PCA (3).

### Cerebrospinal fluid analysis

As clinical diagnosis is not confirmed by amyloid PET in 5–38% percent of cases [24–26], we performed subgroup analysis in cases with biomarker evidence of underlying AD pathology to rule out diagnostic uncertainty. A subset of participants (PCA = 18; tAD = 14; controls = 13) provided cerebrospinal fluid for clinical- and/or research purposes. CSF was analysed using Innotest ELISA (Fujirebio Europe N.V., Gent, Belgium), and tau_− 181_ and amyloid-beta_-1-42_(Aβ_1–42_) were measured. CSF profiles of Aβ_1–42_ < 630 ng/L and/or tau_− 181_/Aβ_1–42_ ratio ≥ .88 were considered compatible with AD [27].

### Statistical analysis

#### Power calculation

In a previous meta-analysis, we found pRFNL thinning of 7 μm in 553 AD cases compared to 486 controls, when exclusively selecting SD-OCT scanners [3]. With a true effect of 7 μm and a standard deviation of 8 μm, 21 participants in each group are needed to reject the null hypothesis of no difference between the disease and control group with a power of 0.80. We enrolled > 21 participants in each diagnosis group.

#### Data analysis

Normality of data distribution was visually assessed using histograms and *Q*-*Q* plots. Between-group differences were assessed with one-way ANOVA and Chi-Squared tests for measures that were normally distributed or binary respectively. Linear regression models were used to assess whether changes in retinal (layer) thickness were attributable to diagnosis adjusting for age and sex. All reported beta coefficients (*β*) are standardized *β*. Level of significance for all test was *p* = 0.05, using two-sided tests. Data analysis was performed with IBM SPSS Statistics (version 22.0), and GraphPad Prism (version 6.0) was used to generate graphs.

## Results

Table 1 includes summaries of demographic, retinal measures in the overall cohort and CSF and neuro-imaging biomarkers of sub cohorts.

### Retinal (layer) thickness does not discriminate between disease groups in the overall cohort

**Fig. 2:**
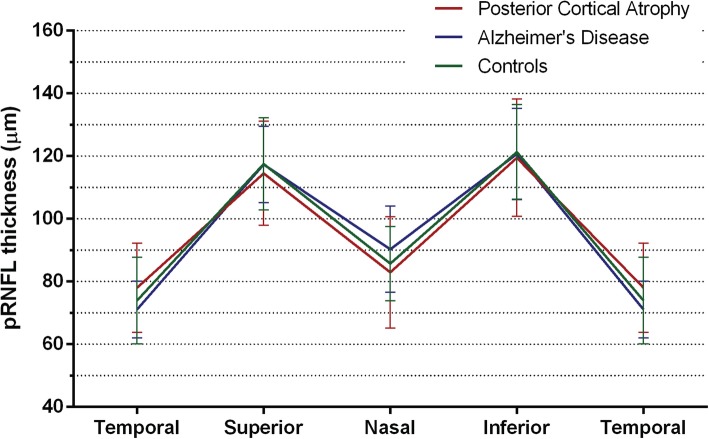
Peripapillary retinal nerve fiber layer thickness. Peripapillary retinal nerve fiber layer (pRNFL) thickness in μm in posterior cortical atrophy (PCA, *n* = 25, red), typical Alzheimer’s disease (tAD, *n* = 23, blue) and controls (*n* = 70, green) (means and SD) in a TSNIT plot showing pRNFL thickness in different sectors (temporal, superior, nasal, inferior)

Overall cohort analysis did not find evidence that patient and control groups differed on any pRNFL measure: total mean; sectoral: temporal, superior, nasal and inferior (one-way ANOVA, all *p* > 0.17) (Fig. 2). Furthermore, overall cohort macular analysis also did not provide evidence that patient and control groups differed on measures of mean mRT and mRT in the fovea, the inner ring, and outer ring of the ETDRS grid (one-way ANOVA, all *p* > 0.65) (Table 2). There was no evidence that signal to noise ratios (SNR) differed between groups for pRNFL ring scans (one-way ANOVA, *F* (2, 115) = 0.81, *p* = 0.45) or macular measures (one-way ANOVA, *F* (2, 107) = 0.89, *p* = 0.41).

**Table 2:** Macular retinal thickness in the overall cohort for PCA, tAD and control groups

	Posterior cortical atrophy (*n* = 23)	Typical Alzheimer’s disease (*n* = 22)	Controls (*n* = 66)	PCA		tAD	
				Beta	*p* value	Beta	*p* value
Total macular retinal thickness (mRT)	266.9 (±16.3)	267.8 (±13.6)	269.3 (±13.6)	− 0.07	0.50	− 0.09	0.38
Fovea	223.0 (±19.1)	227.7 (±21.5)	224.3 (±23.8)	− 0.02	0.80	0.01	0.89
Superior inner	286.8 (±20.3)	289.6 (±15.9)	290.0 (±18.9)	− 0.07	0.49	− 0.06	0.56
Temporal inner	276.0 (±17.0)	278.2 (±12.1)	276.0 (±17.4)	0.00	1.00	0.01	0.92
Inferior inner	287.8 (±18.3)	288.0 (±11.5)	289.6 (±17.2)	− 0.04	0.68	− 0.09	0.35
Nasal inferior	286.5 (±19.8)	287.9 (±15.1)	289.5 (±19.8)	− 0.06	0.53	− 0.08	0.38
Superior outer	263.4 (±18.2)	269.3 (±20.4)	268.4 (±15.5)	− 0.12	0.23	− 0.01	0.89
Temporal outer	246.2 (±15.9)	246.8 (±13.9)	247.7 (±14.8)	− 0.04	0.67	− 0.06	0.57
Inferior outer	259.0 (±19.8)	258.6 (±14.5)	261.3 (±15.2)	− 0.05	0.60	− 0.09	0.35
Nasal outer	281.3 (±17.8)	280.5 (±16.7)	285.0 (±16.1)	− 0.08	0.40	− 0.14	0.15
Signal to noise ratio	8.6 (±0.8)	8.6 (±0.7)	8.4 (±0.8)	0.11	0.26	0.10	0.34

Total macular retinal thickness (mRT) in Early Treatment in Diabetes Retinopathy Study (ETDRS) regions in posterior cortical atrophy (PCA), typical Alzheimer’s disease (tAD) and control participants. Means (±SD) are shown together with standardized betas and *p*-values from linear regression models with macular measures as dependent variable and diagnosis as an independent variable, adjusted for age and sex

Regression analyses adjusting for age and gender did not find evidence that patient and control groups differed on pRNFL or mRT measures (Table 1). While there were significant associations between age and measures of retinal (layer) thickness (pRNFL: *β* = − 0.28, *p* < 0.01; mRT: *β* = − 0.26, p < 0.01), there were no statistically significant associations between MMSE and measures of retinal (layer) thickness (pRNFL: *β* = 0.001; *p* = 0.94; mRT: *β* = 0.10, *p* = 0.29).

### Retinal (layer) thickness does not discriminate between groups in MRI and CSF sub cohorts

Table 1 shows comparisons of cortical thickness, subcortical volumes and CSF measures between PCA, tAD and control groups.

### MRI sub cohort

**Fig. 3:**
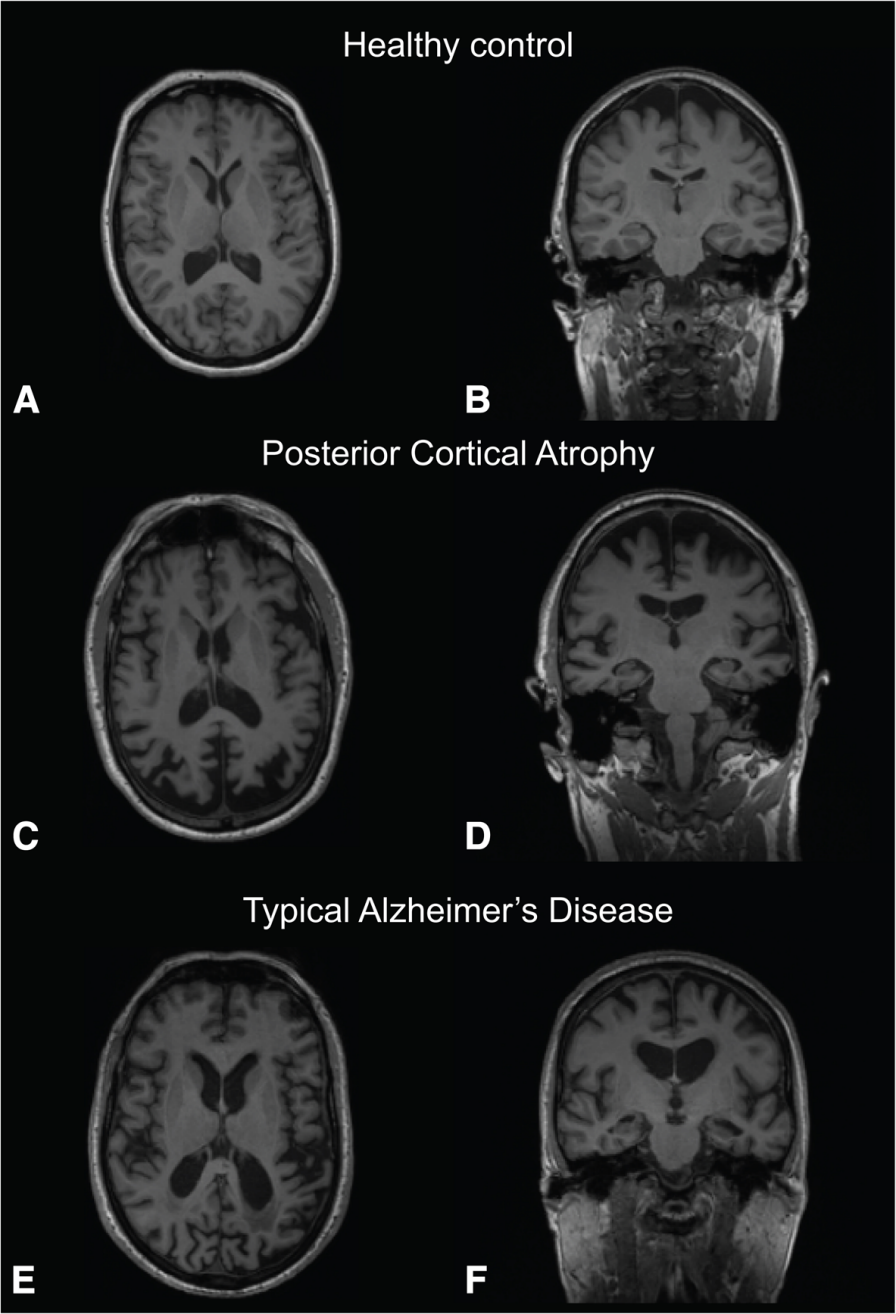
Neurodegenerative features on T1 MRI. Transverse (**a**, **c**, **e**) and coronal (**b**, **d**, **f**) representative T1-weighted volumetric MRI scans in a control, a posterior cortical atrophy (PCA) and a typical Alzheimer’s disease (tAD) case (left hemisphere is shown on the right and vice versa). The PCA case shows predominant parietal-occipital atrophy (**c**) and relatively preserved hippocampal volume (**d**) while the tAD case shows evidence of more widespread neocortical atrophy, although with relatively preserved occipital volume (**e**) and more extensive hippocampal volume loss (**f**)

Visual assessment of available T1 MRI scans showed predominant parietal-occipital cortical atrophy in PCA cases, parietal-temporal cortical and hippocampal atrophy in AD cases, while controls showed no signs of cortical atrophy. Figure 3 shows an example of neurodegenerative features in representative PCA and tAD cases. Quantitative assessment of cortical thickness and subcortical volumes using Freesurfer software found cortical thinning of AD signature thickness and decreased bilateral hippocampal volume in tAD and PCA relative to controls groups, but not different in tAD compared to PCA (one-way ANOVA both *p* < 0.001, post hoc Tukey *p* = 0.17). There was greater cortical thinning in the PCA signature region in PCA relative to tAD and control groups (one-way ANOVA *p* < 0.001 and post hoc Tukey *p* < 0.001).

Despite clear group differences on MRI measures, retinal measures in the MRI sub cohort found no evidence of between-group differences in mean pRNFL and mRT measures (one-way ANOVA, *F* (2, 63) = 0.29, *p* = 0.75, and *F* (2, 63) = 0.61, *p* = 0.55, respectively).

### CSF analysis sub cohort

All PCA and tAD cases met criteria for a CSF AD profile [27] (Table 1). Assessing retinal measures in the CSF sub cohort found no evidence of between-group differences in mean pRNFL and mRT measures (one-way ANOVA *F* (2, 41) = 1.23, *p* = 0.30 and *F* (2, 38) = 0.39, *p* = 0.68 respectively). There were no statistically significant associations between mean pRNFL and mean mRT with CSF amyloid-beta (pRNFL: *β* = − 0.01, *p* = 0.94; mRT: *β* = − 0.13, *p* = 0.41) and tau levels (pRNFL: *β* = 0.13, *p* = 0.59; mRT *β* = 0.05, *p* = 0.76).

## Discussion

In this study, we assessed retinal (layer) thickness measured with OCT as a non-invasive biomarker for neurodegeneration in PCA and tAD compared to control participants. To our knowledge, we are the first to report retinal thickness in PCA cases, the most common atypical phenotype of AD preferentially affecting parietal-occipital cortices involved in visual processing [10]. In contrast to earlier studies in AD, we found no evidence of overall differences in retinal thickness between controls and either patient group. Our findings thus strongly question the utility of cross-sectional retinal thickness measurements with OCT as a diagnostic biomarker of AD.

This sample represents one of the largest, well-characterized samples of both PCA and tAD that includes OCT imaging. As PCA primarily affects cortices involved in visual processing, this cohort is well positioned to test the hypothesis of retrograde atrophy originating from visual cortices. In addition to fulfilling clinical criteria, we performed additional analysis on patients with quantified neurodegeneration and patients with a CSF profile consistent with AD, to provide insight regarding effect sizes of retinal versus established biomarkers. Consistent with our findings in the overall cohort, our subgroup analysis showed that despite unequivocal neurodegeneration on MRI and amyloid positivity based on CSF, retinal (layer) thinning was not observed in tAD and PCA.

There are a number of considerations for the interpretation of results from the current study, as they were inconsistent with earlier studies showing retinal (layer) thinning in AD [3]. In the current cohort, both tAD and PCA patients were relatively young (mean age 65 years), as compared to previous studies (mean age 74 years) [3]. Despite similar underlying proteinopathies (β-amyloid, tau), clinico-radiological differences exist between patients with early and late onset AD (EOAD, LOAD) [28]. For example, atypical presentations are more common in EOAD (33% vs. 6%) and cortical atrophy patterns differ [28–30]. We previously found that retinal thickness did not discriminate EOAD from controls in a well-characterized sample from an independent center [31], consistent with other studies involving EOAD cases [32]. While it is possible that the retina is less affected in EOAD, differences between EOAD and LOAD could also be interpreted as an age effect or a synergistic effect between AD and age in LOAD cases. The current unique cohort enabled assessment of cases with AD pathology with minimal contribution of ageing and age-related comorbidities such as AMD and glaucoma [33]. Indeed, findings include robust associations between age and retinal measures rather than between AD and retinal measures. However, differentiating age from disease effects ultimately requires future studies directly comparing EOAD and LOAD.

Previous study samples have tended to comprise patients at more advanced stages of AD relative to the current sample, based on general measures of disease severity (MMSE score). Retinal thinning may occur late in the disease course, following cortical atrophy. In the current cohort, reliable discrimination of patient from control participants based on MMSE scores, structural neuro-imaging and CSF biomarkers queries the additive diagnostic value of cross-sectional OCT measurements in the clinic in the early to moderate stage of dementia.

The current study used the Optos OCT/SLO device. Direct comparison of different OCT devices is challenging, due to differences in scan area, axial resolution, imaging protocols, and segmentation methods between devices [34]. As our device is a SD-OCT scanner with similar resolution and acquisition times as previously reported OCT scanners, we do not expect this to have influenced results. In addition, as both patients and a large control sample were scanned with the same device, the current lack of evidence for between-group differences cannot be accounted for by discrepancies in measurements.

A limitation of our study is a relatively limited ophthalmological examination of participants, as no detailed eye examination was conducted, and therefore subtle, pre-clinical changes of glaucoma and diabetic retinopathy cannot completely be ruled out. OCT and fundus photographs from all our participants were, however, assessed for ophthalmological comorbidity by an independent ophthalmologist in order to establish any relevant pathology. Secondly, assessment of individual layer thickness of the macula could not be delineated with the OCT system used in this study, ruling out the possibility of establishing that different retinal layers changed in subtle ways without influencing total macular thickness. Future studies should assess the value of measuring individual macular layer thickness, including the RNFL. While representing a limitation of the current study, comparisons of individual or total macular layer thickness between amyloid proven EOAD, LOAD and control participants from the independent Amsterdam Dementia Cohort have not provided evidence of group differences [35]. Thirdly, MRI and CSF subgroup analyses were slightly underpowered to detect pairwise group differences, and conclusions from these analyses should be interpreted with caution. Fourthly, the focus of the current study was on relative biomarker differences in two patient groups compared to a control comparator group. However, particularly given the emergence of atrophy in healthy ageing, future studies might evaluate MRI and OCT measures over time, and study how these relate to purported biomarker group differences either cross-sectionally or longitudinally. Lastly, we did not systemically acquire information on vascular risk factors. As these may influence retinal thickness directly or through diabetes mellitus and hypertensive retinopathy, future studies should consider collecting these, thereby taking vascular risk factors into account as a possible confounder in the relationship between neurodegenerative disease and retinal thinning. Nevertheless, these limitations do not appear to be sufficient to challenge the lack of evidence of retinal (layer) thinning in AD and PCA participants in the presented study.

It is worth noting that retinal (layer) thickness is highly variable among the normal population [36]. Retinal (layer) thinning is a nonspecific finding and is affected by ageing [37], diabetes mellitus [38–43], AMD [44] and glaucoma [45], as well as in other neurodegenerative diseases such as Parkinson’s disease [46] and multiple sclerosis [47]. An additional, possibly small, disease effect from AD is therefore likely to be challenging to detect cross-sectionally. Longitudinal measurements might be more sensitive to detect such small disease effects and ‘normalize’ for interpersonal differences. In addition, future research should focus on molecular imaging of the retina in AD, such as tau, β-amyloid and neuro-inflammation. Molecular biomarkers could have a larger effect size and specificity like in CSF [48], and answer the need for therapeutic read-outs focused on pathological molecular pathways in AD. Presence of these molecular changes in the retina remains controversial, however, and needs confirmation in post-mortem cohorts [7, 49–51]. If proven unequivocally, emerging optical techniques such as fluorescent imaging [52], two-photon microscopy [53], (stimulated) Raman [54] and hyperspectral imaging [55] might be tools to image these molecular changes in the retina in vivo in the future.

## Conclusions

In the current study, we did not find evidence that retinal thickness discriminates well-characterized cases of PCA and tAD from control participants despite unequivocal differences on standard clinical, neuro-imaging and CSF measures. Findings do not support the utility of cross-sectional retinal thickness measurements with OCT as an AD biomarker and underline the need for rigorous validation studies for clinical and scientific purposes. Future studies should also focus on more specific AD retinal biomarkers on a molecular level such as amyloid and tau.

## Data Availability

The datasets used and/or analysed during the current study are available from the corresponding author on reasonable request.

## Abbreviations

AMD: Age-related macular degeneration
CBD: Corticobasal degeneration
CSF: Cerebrospinal fluid
DLB: Dementia with Lewy bodies
EOAD: Early-onset Alzheimer’s disease
ETDRS: Early treatment Diabetic retinopathy study
HC: Healthy control
LOAD: Late-onset Alzheimer’s disease
MMSE: Mini-mental state examination
MRI: Magnetic resonance imaging
mRT: Total macular thickness
NIA-AA: National Institute of Aging and Alzheimer’s Association
OCT: Optical coherence tomography
PCA: Posterior cortical atrophy
PET: Positron emission tomography
pRNFL: Peripapillary retinal nerve fiber layer
QC: Quality control
ROI: Region of interest
SD-OCT: Spectral domain optical coherence tomography
SLO: Scanner laser ophthalmoscope
SNR: Signal to noise ratio
tAD: Typical Alzheimer’s disease
UCL: University College London
